# Supplementary material for: Timeliness of routine childhood vaccination in 103 low-and middle-income countries, 1978–2021: A scoping review to map measurement and methodological gaps
Source: PLOS Glob Public Health. 2022 Jul 14;2(7):e0000325. doi: 10.1371/journal.pgph.0000325 (PMC10021799; doi:10.1371/journal.pgph.0000325)
Supplement: S1 Table — (DOCX) [file pgph.0000325.s001.docx]

# S1 Table: Full search strategy in MEDLINE (Ovid)

Search conducted on 01 July, 2021.

| **Search** | **Query** | **Records retrieved** |
| --- | --- | --- |
| #1 (Childhood) | Child, preschool[Mesh] OR exp Infant[Mesh] OR infant*[tw] OR child*[tw] OR babies[tw] OR newborn*[tw] | 3,208,329 |
| #2 (Vaccination) | Immunization[Mesh] OR immunization schedule[Mesh] OR vaccination[Mesh] OR mass vaccination[Mesh] OR vaccin*[tw] OR immuni#ation*[tw] OR EPI[tw] | 495,076 |
| #3 (Timeliness) | Time Factors[Mesh] OR timeliness[tw] OR timing*[tw] OR delay*[tw] OR age-appropriate[tw] OR “on time”[tw] OR untimely[tw] OR timely[tw] | 1,898,402 |
| #4  (LMICs) | Developing Countries/ OR ((developing or less* developed or under developed or underdeveloped or middle income or low* income) adj (economy or economies))[tiab] OR ((developing or less* developed or under developed or underdeveloped or middle income or low* income or underserved or under served or deprived or poor*) adj (countr* or nation? or population? or world))[tiab] OR (low* adj (gdp or gnp or gross domestic or gross national))[tiab] OR (low adj3 middle adj3 countr*)[tiab] OR (lmic or lmics or third world or lami countr*)[tiab] OR transitional countr*[tiab] OR global south[tiab] OR Democratic People's Republic of Korea"/ OR (North Korea or (Democratic People* Republic adj2 Korea))[tiab] OR Cambodia/ OR Cambodia[tiab] OR Indonesia/ OR (Indonesia or Dutch East Indies)[tiab] OR (Kiribati or Gilbert Islands or Phoenix Islands or Line Islands)[tiab] OR Laos/ OR (Laos or (Lao adj1 Democratic Republic))[tiab] OR Micronesia/ OR Micronesia[tiab] OR Mongolia/ OR Mongolia[tiab] OR Myanmar/ OR (Myanmar or Burma)[tiab] OR Papua New Guinea/ OR (Papua New Guinea or German New Guinea or British New Guinea or Territory of Papua)[tiab] OR Philippines/ OR (Philippines or Philippine Islands)[tiab] OR Solomon Islands[tiab] OR Timor-Leste/ OR (Timor-Leste or East Timor or Portuguese Timor)[tiab] OR Vanuatu/ OR (Vanuatu or New Hebrides)[tiab] OR Vietnam/ OR (Viet Nam or Vietnam or French Indochina)[tiab] OR American Samoa/ OR American Samoa[tiab] OR exp China/ OR China[tiab] OR Fiji/ OR Fiji[tiab] OR Malaysia/ OR (Malaysia or Malayan Union or Malaya)[tiab] OR Marshall Islands[tiab] OR Nauru.t[iab] OR "Independent State of Samoa"/ OR ((Samoa not American Samoa) or Western Samoa or Navigator Islands or Samoan Islands)[tiab] OR Thailand/ OR (Thailand or Siam)[tiab] OR Tonga/ OR Tonga[tiab] OR (Tuvalu or Ellice Islands)[tiab] OR Melanesia/ OR Melanesia[tiab] OR Polynesia/ OR Polynesia[tiab] OR Kyrgyzstan/ OR Kyrgyzstan or Kyrgyz Republic or Kirghizia or Kirghiz)[tiab] OR Moldova/ OR Moldova[tiab] OR Ukraine/ OR Ukraine[tiab] OR Uzbekistan/ OR Uzbekistan[tiab] OR Albania/ OR Albania[tiab] OR Armenia/ OR Armenia[tiab] OR Azerbaijan/ OR Azerbaijan[tiab] OR "Republic of Belarus"/ OR (Belarus or Byelarus or Byelorussia or Belorussia)[tiab] OR Bosnia-Herzegovina/ OR (Bosnia or Herzegovina)[tiab] OR Bulgaria/ OR Bulgaria[tiab] OR "Georgia (Republic)"/ OR Georgia[tiab] not Georgia/ OR Kazakhstan/ OR (Kazakhstan or Kazakh)[tiab] OR Kosovo/ OR Kosovo[tiab] OR Montenegro/ OR Montenegro[tiab] OR "Republic of North Macedonia"/ OR North Macedonia[tiab] OR Romania/ OR Romania[tiab] OR exp Russia/ OR "Russia (Pre-1917)"/ OR USSR/ OR (Russia or Russian Federation or USSR or Union of Soviet Socialist Republics or Soviet Union)[tiab] OR Serbia/ OR Serbia[tiab] OR Turkey/ OR (Turkey.[tiab] not animal/) or (Anatolia or Asia Minor)[tiab] OR Turkmenistan/ OR Turkmenistan[tiab] OR Tajikistan/ OR Tajikistan[tiab] OR Asia, Central/ OR Asia, Northern/ OR Central Asia[tiab] OR Haiti/ OR (Haiti or Hayti)[tiab] OR Bolivia/ OR Bolivia[tiab] OR El Salvador/ OR El Salvador[tiab] OR Honduras/ OR Honduras[tiab] OR Nicaragua/ OR Nicaragua[tiab] OR Argentina/ OR (Argentina or Argentine Republic)[tiab] OR Belize/ OR Belize or British Honduras)[tiab] OR Brazil/ OR Brazil[tiab] OR Colombia/ OR Colombia[tiab] OR Costa Rica/ OR Costa Rica[tiab] OR Cuba/ OR Cuba[tiab] OR Dominica/ OR Dominica[tiab] OR Dominica[tiab] OR Dominican Republic/ OR Dominican Republic[tiab] OR Ecuador/ OR Ecuador[tiab] OR Grenada/ OR Grenada[tiab] OR Guatemala/ OR Guatemala[tiab] OR Guyana/ OR (Guyana or British Guiana)[tiab] OR Jamaica/ OR Jamaica[tiab] OR Mexico/ OR (Mexico or United Mexican States)[tiab] OR Paraguay/ OR Paraguay.mp OR Peru/ OR Peru[tiab] OR Saint Lucia/ OR (St Lucia or Saint Lucia or Iyonala or Hewanorra)[tiab] OR "Saint Vincent and the Grenadines"/ OR (Saint Vincent or St Vincent or Grenadines)[tiab] OR Suriname/ OR (Suriname or Dutch Guiana)[tiab] OR Venezuela/ OR Venezuela[tiab] OR Djibouti/ OR (Djibouti or French Somaliland)[tiab] OR Egypt/ OR Egypt[tiab] OR Morocco/ OR Morocco[tiab] OR Tunisia/ OR Tunisia.mp OR (Gaza or West Bank or Palestine)[tiab] OR Algeria/ OR Algeria[tiab] OR Iran/ OR (Iran or Persia)[tiab] OR Iraq/ OR (Iraq or Mesopotamia)[tiab] OR Jordan/ OR Jordan[tiab] OR Lebanon/ OR (Lebanon or Lebanese Republic)[tiab] OR Libya/ OR Libya[tiab] Or Syria/ OR (Syria or Syrian Arab Republic)[tiab] OR Yemen/ OR Yemen[tiab] OR Afghanistan/ OR Afghanistan[tiab] OR Nepal/ OR Nepal[tiab] OR Bangladesh/ OR Bangladesh[tiab] OR Bhutan/ OR Bhutan[tiab] OR exp India/ OR India[tiab] OR Pakistan/ OR Pakistan[tiab] OR Maldives[tiab] OR Sri Lanka/ OR (Sri Lanka or Ceylon)[tiab] OR Angola/ OR Angola[tiab] OR Cameroon/ OR (Cameroon or Kamerun or Cameroun)[tiab] OR Cape Verde/ Or (Cape Verde or Cabo Verde)[tiab] OR Comoros/ OR (Comoros or Glorioso Islands or Mayotte)[tiab] OR Congo/ OR (Congo not ((Democratic Republic adj3 Congo) or congo red or crimean-congo))[tiab] OR Cote d’Ivoire/ OR Cote d'Ivoire or Cote dIvoire or Ivory Coast)[tiab] OR Eswatini/ OR (eSwatini or Swaziland)[tiab] OR Ghana/ OR (Ghana or Gold Coast)[tiab] OR Kenya/ OR (Kenya or East Africa Protectorate)[tiab] OR Lesotho/ OR (Lesotho or Basutoland)[tiab] OR Mauritania/ OR Mauritania[tiab] OR Nigeria/ OR Nigeria[tiab] OR (Sao Tome abj2 Principe)[tiab] OR Senegal/ OR Senegal[tiab] OR Sudan/ OR (Sudan not South Sudan)[tiab] OR Zambia/ OR Zambia or Northern Rhodesia)[ti,ab] OR Zimbabwe/ OR (Zimbabwe or Southern Rhodesia)[tiab] OR Botswana/ OR (Botswana or Bechuanaland or Kalahari)[tiab] OR Equitorial Guinea/ OR (Equatorial Guinea or Spanish Guinea)[tiab] OR Gabon/ OR (Gabon or Gabonese Republic)[tiab] OR Mauritius/ OR (Mauritius or Agalega Islands)[tiab] OR Namibia/ OR (Namibia or German South West Africa)[tiab] OR South Africa/ OR (South Africa or Cape Colony or British Bechuanaland or Boer Republics or Zululand or Transvaal or Natalia Republic or Orange Free State)[tiab] OR Benin/ OR (Benin or Dahomey)[tiab] OR Burkna Faso/ OR (Burkina Faso or Burkina Fasso or Upper Volta)[tiab] OR Burundi/ OR (Burundi or Ruanda-Urundi)[tiab] OR Central African Republic/ OR (Central African Republic or Ubangi-Shari)[tiab] OR Chad/ OR Chad[tiab] OR "Democratic Republic of the Congo"/ OR (((Democratic Republic or DR) adj2 Congo) or Congo-Kinshasa or Belgian Congo or Zaire or Congo Free State)[tiab] OR Eritrea/ OR Eritrea[tiab] OR Ethiopia/ OR (Ethiopia or Abyssinia)[tiab] OR Gambia/ OR Gambia[tiab] OR Guinea/ OR (Guinea not (New Guinea or Guinea Pig* or Guinea Fowl or Guinea-Bissau or Portuguese Guinea or Equatorial Guinea))[tiab] OR Guinea-Bissau/ OR (Guinea-Bissau or Portuguese Guinea)[tiab] OR Liberia/ OR Liberia[tiab] OR Madagascar/ OR (Madagascar or Malagasy Republic)[tiab] OR Malawi/ OR (Malawi or Nyasaland)[tiab] OR Mali/ OR Mali[tiab] OR Mozambique/ OR (Mozambique or Mocambique or Portuguese East Africa)[tiab] OR Niger/ OR (Niger not (Aspergillus or Peptococcus or Schizothorax or Cruciferae or Gobius or Lasius or Agelastes or Melanosuchus or radish or Parastromateus or Orius or Apergillus or Parastromateus or Stomoxys))[tiab] OR Rwanda/ OR (Rwanda or Ruanda)[tiab] OR Sierra Leone/ OR (Sierra Leone or Salone)[tiab] OR Somalia/ OR (Somalia or Somaliland)[tiab] OR South Sudan/ OR South Sudan[tiab] OR Tanzania/ OR (Tanzania or Tanganyika or Zanzibar)[tiab] OR Togo/ OR (Togo or Togolese Republic or Togoland)[tiab] OR Uganda/ OR Uganda[tiab] OR "africa south of the sahara"/ OR africa, central/ OR africa, eastern/ OR africa, southern/ OR africa, western/ OR ("Africa South of the Sahara" or sub-Saharan Africa or subSaharan Africa)[tiab] OR Central Africa[tiab] OR Eastern Africa[tiab] OR Southern Africa[tiab] OR Western Africa[tiab] | 1,575,940 |
|  | #1 AND #2 AND #3 AND #4 | 2,463 |
| **Limited to**: 1978 – 2021, English and French language. | | **2,153** |
