# Supplementary material for: Timeliness of routine childhood vaccination in 103 low-and middle-income countries, 1978–2021: A scoping review to map measurement and methodological gaps
Source: PLOS Glob Public Health. 2022 Jul 14;2(7):e0000325. doi: 10.1371/journal.pgph.0000325 (PMC10021799; doi:10.1371/journal.pgph.0000325)
Supplement: S2 Table — (DOCX) [file pgph.0000325.s002.docx]

**S2 Table: Summary characteristics of included studies**

| **Author (s)** | **Year**  **published** | **Low-and middle-income country studied** | **Age group**  **studied** | **Study methodology** | **Dataset used for the analysis** |
| --- | --- | --- | --- | --- | --- |
| Abidin et al | 2017 | Malaysia | 0-5 years | cross sectional | facility-based |
| Adetifa et al | 2018 | Kenya | 12-23 months | cross sectional | community-based |
| Agopian et al | 2020 | Armenia | 0-35months | cross sectional | community-based |
| Akmatov et al | 2015 | Burkina Faso, Tanzania Benin, Burundi, Cameroon, Chad, Congo Democratic Republic, Côte d’Ivoire, Ethiopia, Gabon, Ghana, Guinea, Kenya, Lesotho, Liberia, Malawi, Mali, Mozambique, Namibia, Niger, Nigeria, Rwanda, Sao Tome and Principe, Senegal, Sierra Leone, Swaziland, Uganda, Zambia Congo Zimbabwe | 0-5 years | cross sectional | community-based |
| Akmatov et al | 2012 | Albania, Bangladesh, Belarus, Belize, Bosnia and Herzegovina, Burkina Faso, Burundi, Cameroon, Cote d’Ivoire, Djibouti, Gambia, Ghana, Guinea-Bissau, Guy ana, Iraq, Jamaica, Kazakhstan, Laos, Macedonia, Malawi, Mauritania, Mongolia, Montenegro, Serbia, Sierra Leone, Syria, Thailand, Togo, Trinidad and Tobago, Vietnam, Yemen | 0-59 months | cross sectional | community-based |
| Alam et al | 2021 | Bangladesh | unclear | cross sectional | facility-based |
| Ali et al | 2019 | Iraq | 1-2 years | cross sectional | facility-based |
| Alkoshi et al | 2020 | Libya | 0-18 months | cross sectional | facility-based |
| Alrowali et al | 2019 | Saudi Arabia | 0-23 months | cross sectional | facility-based |
| Al-shemari et al | 2006 | Iraq | 0-48 months | cross sectional | facility-based |
| Alsuhaibani | 2020 | Saudi Arabia | 0-24months | cross sectional | community-based |
| An et al | 2016 | Vietnam | 0-5 years | cross sectional | community-based |
| Anbesu et al | 2021 | Ethiopia | 0-11 months | cross sectional | community-based |
| Ateudjieu et al | 2020 | Cameroon | 0-59 months | cross sectional | facility-based |
| Awafeso et al | 2013 | India | 12-23 months | cross sectional | community-based |
| Babirye et al | 2012 | Uganda | 10-23 months | cross sectional | community-based |
| Bangure et al | 2015 | Zimbabwe | unclear | RCT | facility-based |
| Banjari et al | 2018 | Saudi Arabia | 0-35 months | cross sectional | facility-based |
| Banwat et al | 2014 | Nigeria | 0-12 months | RCT | community-based |
| Barman et al | 2015 | India | 12-36 months | cross sectional | community-based |
| Belmar-george | 2018 | Saint Lucia | 9-14 years | cross sectional | community-based |
| Bicaba et al | 2009 | Burkina Faso | 0-23 months | cross sectional | community-based |
| Bondo et al | 2018 | Malawi | 2 - 16 months | cross sectional | facility-based |
| Borus et al | 2004 | Kenya | 0-2 years | cross sectional | facility-based |
| Boulton et al | 2019 | Ethiopia | 1-5 years | cross sectional | community-based |
| Calhoum et al | 2014 | Kenya | 12-23 months | cross sectional | community-based |
| Chen et al | 2019 | Uganda | 0-71 months | cross sectional | facility-based |
| Chiabi et al | 2017 | Cameroon | 0-11 months | cross sectional | facility-based |
| Choudhary et al | 2018 | India | 6-11 months | case-control | community-based |
| Choudhary et al | 2019 | India | 10 -23 months | cross sectional | community-based |
| Chung et al | 2016 | China | > 12 months | cross sectional | community-based |
| Clark et al | 2009 | Bangladesh, Benin, Bolivia, Brazil, Burkina Faso, Cambodia, Cameroon, Chad, Colombia, Comoros, Congo, Côte d'Ivoire, Dominican Republic, Egypt, Eritrea, Gabon, Ghana, Guatemala, Guinea, Haiti, Honduras, India, Kenya, Kyrgyz Republic, Lesotho, Madagascar, Malawi, Mali, Mauritania, Morocco, Mozambique, Namibia, Nicaragua, Niger, Nigeria, Peru, Rwanda, Senegal, Tanzania, Togo, Turkey, Uganda, Uzbekistan, Yemen, Zambia | 0-5 years | cross sectional | community-based |
| Corsi et al | 2009 | India | 0-5 years | cross sectional | community-based |
| Cui et al | 2010 | China | unclear | cross sectional | community-based |
| Cui et al | 2007 | China | 12-23 months | cross sectional | community-based |
| Cutts et al | 1991 | Guinea, Mozambique | 12-23 months | cross sectional | community-based |
| Danjuma et al | 2020 | Nigeria | newborns | cross sectional | facility-based |
| D'ardenne et al | 2016 | Guatemala, Peru | 0-5 years | cross sectional | community-based |
| Datar et al | 2005 | India | 2-35 months | cross sectional | community-based |
| Dayan et al | 2006 | Argentina | 13-59 months | cross sectional | community-based |
| Delrieu et al | 2015 | Burkina Faso, Ghana, Kenya, Senegal, Tanzania | 0-5 years | cross sectional | community-based |
| Dionne-odom et al | 2018 | Cameroon | 12-60 months | cross sectional | community-based |
| Domek et al | 2019 | Guatemala | 2 - 6 months | RCT | facility-based |
| Edstam et al | 2002 | Mongolia | 2 years | cross sectional | facility-based |
| Ettarh et al | 2012 | Kenya | 9-59 months | cross sectional | community-based |
| Fadnes et al | 2011 | South Africa | 0-2 years | RCT | community-based |
| Fadnes et al | 2011 | Uganda | 0-2 years | RCT | community-based |
| Fisker et al | 2014 | Guinea-Bissau | 12-47 months | cohort | community-based |
| Flannery et al | 2013 | Brazil | 19-36 months | cross sectional | community-based |
| Gentile et al | 2015 | Argentina | 6-24 months | cross sectional | facility-based |
| Gibson et al | 2017 | Kenya | 0-12 months | RCT | facility-based |
| Gibson et al | 2015 | Kenya | 12-23 months | cross sectional | community-based |
| Gil et al | 2015 | India | 0-12 months | cross sectional | community-based |
| Giao et al | 2019 | Vietnam | 12-24 months | cross sectional | facility-based |
| Gram et al | 2014 | Ghana | 0-11 months | cross sectional | community-based |
| Gunning et al | 2020 | Zambia | 0-12months | cohort | facility-based |
| Hafele et al | 2020 | Laos | 8-28months | cross sectional | facility-based |
| Han et al | 2014 | China | 12-59 months | cross sectional | community-based |
| Hasanain et al | 2002 | Saudi Arabia | 2-52 months | cross sectional | facility-based |
| He et al | 2021 | China | 1-6 years | cohort | facility-based |
| Hoest et al | 2017 | Bangladesh, Brazil, India, Nepal, Peru, Pakistan, South Africa, Tanzania | 0-24 months | cross sectional | community-based |
| Holambe et al | 2013 | India | infants | cross sectional | facility-based |
| Hu et al | 2017 | China | 6 months - 3 years | cross sectional | HIMS data |
| Hu et al | 2013 | China | 18-48 months | cross sectional | community-based |
| Hu et al | 2017 | China | 24-35 months | cross sectional | community-based |
| Hu et al | 2015 | China | > 12 months | cross sectional | community-based |
| Hu et al | 2018 | China | 24-35 months | cross sectional | community-based |
| Hu et al | 2020 | China | 12-23 months | cross sectional | community-based |
| Hu et al | 2018 | China | 0-26 months | cross sectional | HIMS data |
| Hu et al | 2018 | China | 24-35 months | cross sectional | community-based |
| Hu et al | 2014 | China | > 12 months | cross sectional | community-based |
| Huges et al | 2016 | Nepal | 0-6 months | cohort | community-based |
| Hutin et al | 2013 | China | unclear | cross sectional | community-based |
| Hyunh | 2021 | Vietnam | 12-24 months | cross sectional | facility-based |
| Ibraheem et al | 2019 | Nigeria | 0-12months | cross sectional | facility-based |
| Igarashi et al | 2010 | Zambia | unclear | cross sectional | community-based |
| Jadidi et al | 2015 | Iran | 24-47 months | cohort | community-based |
| Jahn et al | 2008 | Malawi | 0-5 years | cross sectional | community-based |
| Jain et al | 2021 | India | 0-12 months | cross sectional | community-based |
| Janusz et al | 2021 | Angola, Burkina Faso, Benin, Burundi, Congo Democratic Republic, Congo, Cote D’Ivoire, Cameroon, Ethiopia, Gabon, Ghana, Gambia, Guinea, Kenya, Comoros, Liberia, Lesotho, Mali, Malawi, Mozambique, Nigeria, Niger, Namibia, Rwanda, Sierra Leone, Senegal, Chad, Togo, Tanzania, Uganda, South Africa, Zambia, Zimbabwe | 12-35 months | cross sectional | community-based |
| Jones et al | 2021 | Madagascar | 0-23 months | cross sectional | facility-based |
| Kagucia et al | 2021 | Kenya | 0-6 months | RCT | facility-based |
| Kahn et al | 1995 | Central African Republic | 12-23 months | cross sectional | community-based |
| Kaji et al | 2016 | Thailand | migrant children | cohort | school-based |
| Kang et al | 2014 | China | 7-10 months | cross sectional | community-based |
| Kidanne et al | 2019 | Ethiopia | 12-23 months | cross sectional | community-based |
| Kumar et al | 2017 | India | 12-23 months | cross sectional | community-based |
| Kuruvilla et al | 2009 | India | 12-24 months | cross sectional | community-based |
| Kyuregyan et al | 2021 | Russia | 0-12 months | cross sectional | mixed |
| Laryea et al | 2014 | Ghana | 2-28 months | cross sectional | facility-based |
| Laus`evie et al | 2009 | Montenegro | 22-34 months | cross sectional | facility-based |
| Levine et al | 2021 | Ghana | 0-12 months | RCT | community-based |
| Li et al | 2020 | Kenya | 0-23months | cross sectional | community-based |
| Li et al | 2021 | China | 0-6 years | cross sectional | facility-based |
| Li et al | 2014 | China | 1-7 years | cross sectional | HIMS data |
| Li et al | 2017 | China | infants | cross sectional | facility-based |
| Li et al | 2020 | China | 8-24months | cross sectional | facility-based |
| Lin et al | 2014 | China | 9 months - 2 years | case-control | HIMS data |
| Lindqvist et al | 2019 | Sri Lanka | 0-5years | cross sectional | facility-based |
| Liu et al | 2018 | China | 0-35 months | cross sectional | community-based |
| Lopez et al | 2018 | Philippines | 5-6 years | cross sectional | community-based |
| Loy et al | 2020 | Singapore | 0-24months | cohort | HIMS data |
| Lugollo et al | 2008 | Brazil | unclear | case-control | community-based |
| Luz et al | 2016 | Colombia | 6 months -8 years | cross sectional | community-based |
| Mansour et al | 2018 | Lebanon | 12-59 months | cross sectional | community-based |
| Marban-castro et al | 2018 | Mozambique | 0-3years | cross sectional | community-based |
| Marefiaw et al | 2019 | Ethiopia | 12-23 months | cross sectional | community-based |
| Master et al | 2018 | Ethiopia | 3-12 months | cross sectional | community-based |
| Masters et al | 2018 | Kenya | 1-4 years | cross sectional | community-based |
| Mbengue et al | 2017 | Senegal | 12-23 months | cross sectional | community-based |
| Mekonnen et al | 2020 | Ethiopia | 12-23 months | cross sectional | community-based |
| Mekonnen et al | 2021 | Ethiopia | unclear | RCT | facility-based |
| Mensah et al | 2019 | Madagascar | unclear | cross sectional | community-based |
| Miyahara et al | 2016 | Gambia | unclear | cross sectional | community-based |
| Mohammedbeigi et al | 2015 | Iran | 24-47 months | cohort | community-based |
| Mohhtari et al | 2015 | Iran | 24-47 months | cohort | community-based |
| Moisi et al | 2010 | Kenya | unclear | cross sectional | community-based |
| Monrgomery et al | 2015 | China | > 8 months | cross sectional | community-based |
| Moturi et al | 2018 | Botswana, Gambia, Namibia, Nigeria, Sao Tome and Principi | newborns | cross sectional | facility-based |
| Mthiyane et al | 2019 | South Africa | 12-59months | cross sectional | community-based |
| Musa et al | 2021 | Bosnia and Herzegovina | 12-35 months | cross sectional | facility-based |
| Mutua et al | 2015 | Kenya | unclear | cohort | community-based |
| Mutua et al | 2021 | Angola, Benin, Burkina Faso, Burundi, Central African Republic, Cameroon, Chad, Comoros, Congo, Congo Democratic Republic, Cote d'Ivoire, Eswatini, Ethiopia, Gabon, Gambia, Ghana, Guinea, Guinea Bissau, Kenya, Lesotho, Liberia, Madagascar, Malawi, Mali, Mauritania, Mozambique, Namibia, Niger, Nigeria, Rwanda, Sao Tome and Principe | 12-36 months | cross sectional | community-based |
| Mutua et al | 2016 | Kenya | 12-23 months | cohort | community-based |
| Mutua et al | 2020 | Kenya | 12-23 months | cross sectional | community-based |
| Mvula et al | 2016 | Malawi | >6years | cohort | community-based |
| Nadella et al | 2019 | Tanzania | 0-12months | cross sectional | community-based |
| Nakatudde et al | 2019 | Uganda | 6-24 months | cross sectional | facility-based |
| Nalley et al | 2019 | Nigeria | 12-23 months | cross sectional | community-based |
| Narvaez et al | 2017 | Colombia | 0-6 years | cross sectional | community-based |
| Ndiritu et al | 2006 | Kenya | 9-23 months | cross sectional | community-based |
| Ni et al | 2017 | China | 12-72 months | cross sectional | community-based |
| Noh et al | 2019 | Pakistan | unclear | cross sectional | community-based |
| Noh et al | 2018 | Pakistan | 0-23 months | cross sectional | community-based |
| Ochoa et al | 2015 | Peru | 0-12 months | cohort | facility-based |
| Odusanya et al | 2000 | Nigeria | 0-12 months | cross sectional | community-based |
| Odutola et al | 2015 | Gambia | 12-59 months | cross sectional | facility-based |
| Olademije et al | 2020 | Nigeria | 0-10months | RCT | facility-based |
| O'leary et al | 2016 | Ghana | low birthweight | cohort | community-based |
| Olusanta | 2010 | Nigeria | 0-3 months | cross sectional | facility-based |
| Oner et al | 2012 | Turkey | 12-23 months | cross sectional | community-based |
| Ork et al | 2019 | Cambodia | 5-7 years | cross sectional | community-based |
| Oue`draogo et al | 2013 | Burkina Faso | 0-5 years | cross sectional | community-based |
| Parameswaran et al | 2012 | Sri Lanka | 12-23 months | cross sectional | community-based |
| Park et al | 2011 | South Korea | 1-72 months | cross sectional | community-based |
| Park et al | 2013 | South Korea | unclear | cross sectional | facility-based |
| Patel et al | 2014 | Philippines | 0-6 weeks | cross sectional | facility-based |
| Patel et al | 2016 | French Polynesia | 0-6 years | cross sectional | school-based |
| Pe`rie`res et al | 2021 | Senegal | unclear | cross sectional | community-based |
| Perrinho et al | 1987 | South Africa | 12-23 months | cross sectional | community-based |
| Pertet et al | 2018 | Kenya | 0-23 months | cross sectional | community-based |
| Pham et al | 2018 | Vietnam | 6-11 months | cross sectional | community-based |
| Pindyck et al | 2019 | Burkina Faso, Ghana, Rwanda, Zimbabwe | 3-36 months | cross sectional | community-based |
| Poorolajal et al | 2012 | Iran | 12-24 months | cross sectional | community-based |
| Prinja et al | 2009 | India | 0-17 months | cohort | community-based |
| Quazi et al | 2018 | Pakistan | 0-12months | cross sectional | facility-based |
| Raguindin et al | 2021 | Philippines | 0-12 months | cross sectional | facility-based |
| Rainey et al | 2012 | Haiti | 12-23 months | cross sectional | community-based |
| Ramaswamy et al | 2014 | India | 0-12 months | cross sectional | Facility-based |
| Rammohan et al | 2015 | India | 12-60 months | cross sectional | community-based |
| Rammohan et al | 2014 | India | 12-59 months | cross sectional | community-based |
| Rauniyar et al | 2020 | Mongolia | 12-23 months | cross sectional | community-based |
| Rejali et al | 2015 | Iran | 24 - 47 months | cross sectional | community-based |
| Roux et al | 2017 | South Africa | 0-11 months | cohort | community-based |
| Sadoh et al | 2009 | Nigeria | >12 months | cross sectional | facility-based |
| Sadoh et al | 2014 | Nigeria | 2 months - 15 years | cross sectional | facility-based |
| Sadoh et al | 2013 | Nigeria | unclear | cross sectional | facility-based |
| Sahoo et al | 2018 | India | 0-11 months | cross sectional | facility-based |
| Salameh et al | 2021 | Jordan | 0-18 months | cross sectional | facility-based |
| Saraiva et al | 2015 | Brazil | 7-18 months | cross sectional | community-based |
| Sartori et al | 2017 | Brazil | 0-23 months | cohort | HIMS data |
| Sato | 2020 | Nigeria | 12-59months | cross sectional | community-based |
| Schoeps et al | 2014 | Burkina Faso | 12-23 months | cross sectional | community-based |
| Schweitzer et al | 2016 | Honduras | 0-59 months | cross sectional | community-based |
| Schweitzer et al | 2017 | Albania, Armenia, Azerbaijan, Bangladesh, Benin, Bolivia, Burkina Faso, Burundi, Cambodia, Cameroon, Colombia, Comoros, Congo, Côte d’Ivoire, Democratic Republic of the Congo, Egypt, Gabon, Ghana, Guyana, Honduras, Jordan, Kenya, Kyrgyzstan, Lesotho, Liberia, Madagascar, Malawi, Maldives, Mali, Mozambique, Namibia, Niger, Nigeria, Pakistan, Peru, Republic of Moldova, Rwanda, Senegal, Sierra Leone, Swaziland, Tajikistan, Tanzania, Timor-Leste, Uganda, Zambia, Zimbabwe | 12-60 months | cross sectional | community-based |
| Schweitzer et al | 2015 | Armenia, Kyrgyzstan | 0-59 months | cross sectional | community-based |
| Scott et al | 2014 | Gambia | 9-60 months | cross sectional | community-based |
| Senessie et al | 2007 | Sierra Leone | 0-35 months | cross sectional | community-based |
| Sheik et al | 2018 | Bangladesh | 12-23 months | cross sectional | community-based |
| Shrivastwa et al | 2016 | India | 0-60 months | cross sectional | mixed |
| Siddiqi et al | 2010 | Pakistan | unclear | cross sectional | community-based |
| Siddiqi et al | 2007 | Pakistan | 0-11 months | cross sectional | community-based |
| Siddiqi et al | 2020 | Pakistan | 0-12months | RCT | facility-based |
| Singh et alz | 2020 | India | 12-23 months | cross sectional | community-based |
| Soeung et al | 2012 | Cambodia | unclear | cross sectional | facility-based |
| Sood et al | 2015 | India | 12-23 months | cross sectional | community-based |
| Sua`rez-castaneda et al | 2014 | El Salvador | 23-59 months | cross sectional | community-based |
| Subbish et al | 2019 | India | 0-23 months | cross sectional | facility-based |
| Sun et al | 2010 | China | 12-35 months | cross sectional | community-based |
| Tang et al | 2017 | China | 18-54 months | cross sectional | community-based |
| Tang et al | 2021 | China | 18-48 months | cross sectional | community-based |
| Tang et al | 2016 | China | 18-54 months | cross sectional | community-based |
| Tauil et al | 2017 | Brazil | 0-24 months | cohort | facility-based |
| Thysen et al | 2014 | Guinea-Bissau | 12-23 months | cross sectional | community-based |
| Tippins et al | 2017 | Federated States of Micronesia | 24-35 months | cross sectional | community-based |
| Toikilik et al | 2010 | Papua New Guinea | 12-23 months | cross sectional | community-based |
| Tooke et al | 2019 | South Africa | low birthweight | cross sectional | HIMS data |
| Tsega et al | 2016 | Malawi | 12-23 months | cross sectional | community-based |
| Upadhyah et al | 2017 | India | low birthweight | RCT | facility-based |
| Vasudevan et al | 2014 | Bangladesh | 11-18 weeks | RCT | community-based |
| Vasudevan et al | 2020 | Tanzania | 12-23 months | cross sectional | facility-based |
| Vonasek et al | 2016 | Uganda | 0-5 years | cross sectional | community-based |
| Wagner et al | 2014 | China | 8 months - 6 years | cross sectional | community-based |
| Wagner et al | 2019 | India | 0-5 years | cross sectional | mixed |
| Wagner et al | 2016 | China | 0-24 months | cross sectional | HIMS data |
| Wagner et al | 2014 | China | 2-7 years | cohort | HIMS data |
| Wakadha et al | 2013 | Kenya | 0-14 weeks | RCT | facility-based |
| Wallace et al | 2012 | Philippines | 5-7 months | cross sectional | facility-based |
| Wallace et al | 2019 | Indonesia | 0-11months | RCT | facility-based |
| Wambui et al | 2017 | Kenya | 0-23 months | cross sectional | facility-based |
| Wang et al | 2007 | China | 1-20 months | RCT | community-based |
| Waroux et al | 2013 | Tanzania | 0-23 months | cross sectional | community-based |
| Wiesen et al | 2016 | Papua New Guinea | 0-11 months | cross sectional | mixed |
| Wu et al | 2016 | China | 0-24 months | cross sectional | community-based |
| Wu et al | 2017 | China | 6-8 years | cross sectional | community-based |
| Wu et al | 2015 | China | 1-14 years | cross sectional | community-based |
| Xiao et al | 2012 | China | 0-14 years | cross sectional | community-based |
| Yadav et al | 2012 | India | 0-5 years | cross sectional | HIMS data |
| Yang et al | 2021 | China | 8-83 months | cross sectional | community-based |
| Yang et al | 2019 | China | 2 -7 years | cross sectional | HIMS data |
| Zaidi et al | 2014 | Pakistan | 0-5 years | cross sectional | community-based |
| Zhou et al | 2016 | China | unclear | cross sectional | facility-based |
| Zhou et al | 2009 | China | unclear | cross sectional | community-based |
| Zivich et al | 2017 | Democratic Republic of the Congo | 0-6 months | cohort | facility-based |
