# Supplementary material for: Timeliness of routine childhood vaccination in 103 low-and middle-income countries, 1978–2021: A scoping review to map measurement and methodological gaps
Source: PLOS Glob Public Health. 2022 Jul 14;2(7):e0000325. doi: 10.1371/journal.pgph.0000325 (PMC10021799; doi:10.1371/journal.pgph.0000325)
Supplement: S3 Table — (DOCX) [file pgph.0000325.s003.docx]

**S3 Table: List of 46 low-and middle-income countries that were not the focus of a single study but contributed data to the 13 studies that were based on multiple countries**

| **Countries** | **Number of studies** |
| --- | --- |
| Albania | 2 |
| Angola | 2 |
| Azerbaijan | 1 |
| Belarus | 1 |
| Belize | 1 |
| Benin | 5 |
| Bolivia | 2 |
| Botswana | 1 |
| Burundi | 5 |
| Chad | 4 |
| Cote d’Ivoire | 6 |
| Comoros | 4 |
| Congo | 5 |
| Djibouti | 1 |
| Dominican | 1 |
| Egypt | 2 |
| Eritrea | 1 |
| Eswatini | 1 |
| Gabon | 5 |
| Guinea | 5 |
| Guyana | 2 |
| Jamaica | 1 |
| Kazakhstan | 1 |
| Kyrgyzstan | 3 |
| Lesotho | 5 |
| Liberia | 4 |
| Macedonia | 1 |
| Maldives | 1 |
| Mali | 5 |
| Mauritania | 3 |
| Moldova | 1 |
| Morocco | 1 |
| Namibia | 6 |
| Nicaragua | 1 |
| Niger | 4 |
| Rwanda | 6 |
| Sao Tome | 3 |
| Serbia | 1 |
| Swaziland | 2 |
| Syria | 1 |
| Tajikistan | 1 |
| Timor-Leste | 1 |
| Togo | 3 |
| Trinidad and Tobago | 1 |
| Uzbekistan | 1 |
| Yemen | 2 |
