# Supplementary material for: Timeliness of routine childhood vaccination in 103 low-and middle-income countries, 1978–2021: A scoping review to map measurement and methodological gaps
Source: PLOS Glob Public Health. 2022 Jul 14;2(7):e0000325. doi: 10.1371/journal.pgph.0000325 (PMC10021799; doi:10.1371/journal.pgph.0000325)
Supplement: Attachment — Submitted filename: RESPON~1.DOC [file pgph.0000325.s005.DOC]

8^th^ March 2022

Dear Meru Sheel,

Re: Manuscript reference number: PGPH-D-21-01137

**Title: Timeliness of routine childhood vaccination in 103 low-and middle-income countries, 1978 – 2021: a scoping review to map measurement and methodological gaps**

We thank the editors and reviewers for their time in reviewing our manuscript and for these valued comments. We have extensively revised the manuscript in line with these comments and believe that the manuscript is stronger as a result of the review.

We submit a revised manuscript and detailed response to the reviewers that addresses the issues raised by each reviewer.

Yours sincerely,

on behalf of the authors

Dr Oghenebrume Wariri, Dr Uduak Okomo, and Prof Beate Kampmann

# Journal Requirements

|  | **Editor comments** | **Authors’ response** |
| --- | --- | --- |
| 1. | We note that the original search was performed in July 2021. Please discuss whether relevant literature has been published in the interim that would be expected to affect the results of the meta-analysis. | We have discussed the potential impact of the availability of relevant literature on the topic after our search was completed in July 2021.We acknowledge that this is an innate restriction as our study is not a living Systematic Review. We highlighted this as a limitation, on page 12, line 4 – 8. |
| 2. | We have noticed that you have cited figures in the manuscript file. However, there are no corresponding files uploaded to the submission. Please ensure that all files are present to ensure that your paper is fully reviewed. Please provide separate figure files in .tif or .eps format only, and remove any figures embedded in your manuscript file. | We apologise for omitting the figure files during the initial submission. The figure files in .tiff format have now been uploaded with the revised manuscript. |
| 3. | We have noticed that you have uploaded supporting information but you have not included a list of legends.  Please add a full list of legends for all supporting information files (including figures, table and data files) after the references list. | Thank you for bringing this to our attention. We have now included a list of legends for all supporting files at the end of the manuscript, after the references list. |

# Reviewer #1

|  | **Reviewer’s comments** | **Authors’ response** |
| --- | --- | --- |
| 1. | Very well written and clear abstract. The authors could use ‘low-and middle-income’ instead of ‘low-income and middle-income countries (LMICs)’ for consistency. The latter does not match with the title.  The author should follow the journal style to format the manuscript. | We thank the reviewer for the positive comments, and for the time spent reviewing our manuscript. Throughout the manuscript, we have now used the term low-and middle-income countries to ensure consistency.  We have edited the manuscript to ensure it follows the journal style. |
| 2. | Introduction The author could discuss more on the study rationale. It does not answer the “So What” question adequately. | Thank you for raising this important point. In the introduction section, on page 3, lines 31 – 36, we have now added a few sentences to address the issue raised here. |
| 3. | Methodology ‘We refined the initial search strategy after analysing the text words in the title and abstract’-How?. The author could elaborate a bit more on this so that the methodology can be replicated in future. | We have elaborated further on page 5, line 8 on how the initial search strategy was refined. |
| 4. | The search was extended to 2021 to capture up-to-date evidence on the timeliness of routine childhood vaccination. The timeline is not clear. Earlier, you mentioned June 2021. | We have clarified the timeline throughout the manuscript. It now clearly reads July 01,2021 across the manuscript. |
| 5. | Single sentence paragraphs should be avoided. | The manuscript has been edited to ensure that there are no single sentence paragraphs. |
| 6. | Discussion  There are some repetitions of information, such as right and left censoring. The author should interpret this to not sound repetition of results in the discussion section. | The discussion section of the manuscript has been carefully revised to ensure there are no unwarranted repetition of results. |
| 7. | Recommendations  The authors provided recommendations around methodological and programmatic issues; however, at the same time, the author may also discuss policy and program level factors that contributed to the timeliness and some recommendations around that.  For clarity, the author could use a PRISMA flow diagram. | The authors want to thank the reviewer for raising this important point. We have now revised the manuscript on page 13, lines 4-7 to reflect the point raised by the reviewer.  Note that we have now included a PRISMA flowchart which was missing in the original submission. |

# Reviewer #2

|  | **Reviewer’s comments** | **Authors’ response** |
| --- | --- | --- |
| 1. | Thank you for allowing me to review this interesting work. The authors did a good job mapping and summarising the published work on timeliness of routine childhood vaccination in LMICs; methods are sounds and the results are well displayed. I appreciate the methodological distinction made between ‘delayed’, ‘early’ and ‘untimely’ vaccination. I believe the work would benefit from taking the following points into account (further specified below): | We thank the reviewer for these positive comments, and for the time spent reviewing our manuscript. We provide below a point-by-point response to each of the comments raised by the reviewer. |
| 2. | First, I agree with the authors that work is particularly timely and relevant in the context of the COVID-19 pandemic affecting routine vaccination programmes worldwide (including in LMICs). However, the relationship between the pandemic and the timeliness of vaccination is not the aim of this paper and emphasising this link doesn’t do justice to the decades of literature (>200 studies!) that have been summarised in the work. I considered this work an important evidence base for future work investigating the impact of the pandemic on the timeliness of routine vaccination, rather than already measuring the impact itself. We are only just starting to scratch the surface of the impact of the pandemic on routine vaccination… The following recent publication reports on the first effects, and can be referenced in the discussion of this manuscript: <https://doi.org/10.1016/S2214-109X(21)00512-X>. | We agree with the reviewer that this work is an important evidence base for future work investigating the impact of the pandemic on the timeliness of routine vaccination, rather than already measuring the impact itself. The manuscript has been revised to clearly reflect this important point and sentences that appear to make COVID-19 the focus of the manuscript have been edited out in the abstract section on page 2, lines 23-24; and on page 3, lines 7, and 9 of the introduction section. We have added additional sentences in the discussion section referencing the potential impact of the ongoing pandemic on vaccination timeliness in the near future. |
| 3. | Second, regarding the methods, the choice for the current design and its methods (scoping review – still unknown to many) can be explained and justified with a bit more detail. Importantly, the decision to restrict to peer-reviewed work in English and French only needs to be well justified, if not reconsidered, as I believe including at least official reports from national (public health) institutes and inclusion of other languages would be a valuable addition to this work | A few sentences (Page 4, lines 10 – 18) have been added to the materials and methods section which justifies the use of the scoping review approach. It provides some details about the utility of scoping reviews, for example, in examining research gaps and compared it with systematic reviews, which are more common.  The decision to only include peer-reviewed articles has been justified in the methods section on page 5, lines 24 – 26. |
| 4. | After revising the manuscript taking the comments presented below (and those of other reviewers) into account, I consider this work a valuable addition to the literature, of interest to the readership of PLOS GPH. | Thank you for this kind comment. |
| 5. | Key words: add ‘scoping review | Done |
| 6. | Abstract - methods: specify “recognised scoping review approaches” | The manuscript has been edited to reflect the requested revision. The sentence now reads *‘’..following the guidance framework for scoping reviews by the Joanna Briggs Institute (JBI)’’* |
| 7. | Abstract: Revise the conclusion with something informative, as the current statement is very generic: “Our review provides evidence for key methodological gaps in the literature when reporting timeliness of childhood vaccination and develops recommendations that could shape the design and implementation of future research.” | The conclusion of the abstract has been revised accordingly. It now reads ‘*’Our findings show that there are fundamental gaps related to the design, conduct, and reporting of studies on the timeliness of routine vaccination in LMICs which limit, to a great extent, their comparability and utility’’* |
| 8. | Abstract & Introduction – first paragraph: In line with my first general comments on COVID-19 and this work; please take the statement in the abstract “Despite its detrimental effect on uptake of childhood vaccination, to date, only one study published so far, has explored the potential impact of COVID-19 on vaccination timeliness.” out, because it is not surprising that not much has been published yet. The same applied for the first paragraph of the introduction: move the focus away from the pandemic and put the pandemic in the context of other factors contributing to timeliness of routine childhood vaccination (e.g. war, vaccine hesitancy). | The manuscript has been revised to clearly reflect this important point. The sentences that appear to make COVID-19 the focus of the manuscript have been edited out in the abstract section on page 2, lines 23-24; and on page 3, lines 7, and 9 of the introduction section. |
| 9. | Introduction – last paragraph: please stick to the aim and objectives of the study and move the details to the method section of the work. Especially panel 1 belongs in the methods, not the introduction. Please add some argumentation for the chosen methods to help answer the research question: why did you decide to do a scoping review? Especially because this review method is close to the more well-known systematic literature review, it would be good to clarify this early (in the introduction), as well as in the methods. | A new paragraph (Page 4, lines 10 – 18) has been added to the materials and methods section that justifies the use of the scoping review approach. It provides some details about the utility of scoping reviews, for example, in examining research gaps and compared it with systematic reviews, which are more common.  Box 1 has also been moved to the materials and methods section as suggested. |
| 10. | Methods – clarify and justify the choice of study design (scoping review). It is currently not clear how the study differs from its sibling-design, the systematic review. | The choice of a scoping review has been clarified and on page 4, lines 10 – 18. |
| 11. | Methods - I very much appreciate the fact that the study protocol was published ahead of this submitted work, open access: <https://doi.org/10.1371/journal.pone.0253423> | We appreciate your kind comment. |
| 12. | Methods - Reporting guidelines: the appropriate PRISMA-ScR checklist has been provided. | Thank you for this comment. |
| 13. | Methods - I very much appreciate the acknowledgement of the PROSPERO registry, and fact that the study protocol was published ahead of this submitted work, open access: <https://doi.org/10.1371/journal.pone.0253423> | The authors appreciate this kind comment |
| 14. | Methods/discussion – grey literature: because not all countries and institutes report their data on vaccination timeliness in peer-reviewed literature (but in reports, e.g. by national public health institutes), I wonder to what extend data has been missed and why the authors decided to include peer-reviewed literature only? This should be discussed as a limitation of the review. | The decision to only include peer-reviewed articles and not grey literature has been justified in the methods section on page 5, lines 24 – 26. This has also been discussed as a potential limitation of our review |
| 15. | Methods/discussion – languages: why published in English and French only? How many studies were excluded based on this language requirement? This should be discussed as a limitation of the review. | The potential implication of excluding studies not published in English or French has been discussed as a potential limitation of this review. |
| 16. | Methods – typo/revise: this sentence doesn’t work “using a thematic using map. | Thank you for spotting this typographical error. The sentence has been duly revised. |
| 17. | Methods/results: it is not clear why a high number (17) of full-texts that were considered eligible based on title and abstract were not available (e.g. paywall). Did the authors contact the authors and ask them for the full text? | We have now provided additional information on page 6, lines 31-32 which clarifies why the 17 articles could not be included. |
| 18. | Supplemental material: please number the respective supplements to make sure they are easy to find. | The supplementary material has been appropriately numbered. It is uploaded as a single file (supplementary file) but has a table of contents that makes it easy to locate specific information. |
| 19. | Results: well presented!  Discussion: well written overall | Thank you for this kind comment |
| 20. | Discussion - limitations: Not necessarily limitations, but a choice made by design that can/should be discussed in the context of the study findings (but not as a limitation): „First, we did not include empiric studies from HICs which would have further strengthened our work.“ Please so add the language and literature restrictions as a limitation (see previous comment). | The section on the limitations of our review has been extensively revised (page 12, lines 1 – 8). It now includes limitations related to language restriction, lack of grey literature, and relevant literature that might have been published since our search was completed in July 2022. |
| 21. | Discussion: this recent publication might be relevant to add: <https://doi.org/10.1016/S2214-109X(21)00512-X> | This recent publication has been cited in the discussion section where we discussed the potential impact of COVID-19 on childhood vaccination. |
| 22. | Results/Discussion - context: I wonder if the authors can elaborate on the similarities and differences in the vaccine programmes itself; i.e. which vaccinations are given, when, and through which healthcare structures. | Thank you for raising this point. We have provided additional information in the discussion section, on page 10, lines 21-26 which highlight the similarities and potential differences in vaccine programmes across countries. |

# Reviewer #3

|  | **Reviewer’s comments** | **Authors’ response** |
| --- | --- | --- |
| 1. | Many thanks for a very well written draft manuscript. This has been quite informative for me. While this is a scoping review of existing literature to map measurements and methodological gaps, I feel like one missing element was the mention of immunisation schedules and their role on this issue of timeliness. | The authors want to thank the reviewer for raising this point. We have provided additional pieces of information and strengthened the discussion section on page 10, lines 17-26, which now highlight the role of immunisation schedules on the issue of vaccination timeliness. |
| 2. | I also wonder if you can strengthen the article a little more with a couple of sentences on how more documentation of timeliness outside of just the traditional metrics of performance would inform national policies on infant immunisations. Because the goal maybe to inform practise, but I think ultimately this type of documentation is more to provide country EPI managers with a reason to look into timeliness and the need to put in place policies that support timeliness for vaccinations. | The second paragraph of the introduction on page 3, lines 12 – 26 has been strengthened to include the revision requested here. The section now includes information highlighting how the documentation of timeliness could help national immunisation managers in detecting gaps in the delivery of certain vaccines, and potentially develop strategies to bridge such gaps. |
| 3. | Otherwise this is very informative, and definitely taps into an area that needs further exploration and refinement as the immunisation programs continue to evolve to meet the needs of the communities they serve. | We thank the reviewer for this positive comment, and for the time spent reviewing our manuscript. |
| 4. | I would be interested to see the maps and figures you have associated with the results once ready. | We apologise for omitting the figures during the initial submission. They have now been uploaded with the revised manuscript. |
