# Supplementary material for: Timeliness of routine childhood vaccination in 103 low-and middle-income countries, 1978–2021: A scoping review to map measurement and methodological gaps
Source: PLOS Glob Public Health. 2022 Jul 14;2(7):e0000325. doi: 10.1371/journal.pgph.0000325 (PMC10021799; doi:10.1371/journal.pgph.0000325)
Supplement: Attachment — Submitted filename: RESPON~1.DOC [file pgph.0000325.s006.DOC]

# Response to Reviewer comments

# Reviewer #1

|  | **Reviewer’s comments** | **Authors’ response** |
| --- | --- | --- |
| 1. | The paper might be considered for publication; however, I also think the paper requires minor revisions. | Thank you for reviewing our manuscript again. All the comments raised have been addressed and a point-by-point response to each comment is provided below. |
| 2. | The abstract should be revised to make it more informative. The rationale of the study is missing in the abstract. The abstract needs proofreading to avoid spacing mistakes. The abstract could be benefitted by adding one or two recommendations. In addition to the themes, the authors should add some data and revise the discussion of the abstract based on the data. | The abstract has been extensively revised and proofread as suggested by the reviewer. We have now included the rational for the study on 2, lines 5-6. We have also added a recommendation to the abstract on page 2, lines 33-34. Lastly, we added some data to the results section as recommended by the reviewer. |
| 3. | In the body of the manuscript, the introduction and the methods are well developed. However, under the methods, how another reviewer verified the extracted data should be mentioned explicitly. How the conflicts were resolved should also be mentioned. | On page 6, lines 10-11 we have included information that explicitly shows how the other reviewer verified the extracted data. Additionally, one page 6, lines 2-3 we have included information on how conflicts or disagreements were resolved during the review process. |
| 4. | Figure 4-D the timeline is a bit confusing and requires revisions. | We have a note under the figure legend which provide some clarity on the timelines for the definitions. |
